# Supplementary material for: Predictive Factors for Gait Recovery in Patients Undergoing Total Hip Arthroplasty: A Propensity Score Weighting Analysis
Source: J Clin Med. 2025 Mar 14;14(6):1979. doi: 10.3390/jcm14061979 (PMC11942875; doi:10.3390/jcm14061979)
Supplement: Supplementary file 1 [file jcm-14-01979-s001.zip › jcm-3498274-supplementary.pdf]

Supplementary Table S1. Clinical predictors and rationale for selection

Predictor	Rationale for selection	Supporting references
Age	Younger age is associated with better postoperative recovery and faster functional mobility restoration.	[13–15]
Gender	Males have greater muscle mass and bone density, potentially leading to better functional recovery after THA.	[13,33,34]
Preoperative gait ability	Strong predictor of postoperative mobility; independent preoperative ambulation is linked to better outcomes.	[13,37]
Postoperative hip flexor strength	Hip flexor strength influences gait performance, including walking speed and step length.	[38-41]
Fracture vs. non-fracture classification	Recovery trajectories differ significantly between acute fracture-related THA and elective THA (e.g., osteoarthritis, avascular necrosis).	[20,21]
Duration of gait disturbance	Longer preoperative gait impairment may negatively affect postoperative recovery.	[10,11]
Rehabilitation duration	Duration of rehabilitation influences functional outcomes and postoperative mobility.	[12,19]
